# A comparative analysis of the 2007 and 2017 Italian chikungunya outbreaks and implication for public health response

**DOI:** 10.1371/journal.pntd.0008159

**Published:** 2020-06-11

**Authors:** Beniamino Caputo, Gianluca Russo, Mattia Manica, Francesco Vairo, Piero Poletti, Giorgio Guzzetta, Stefano Merler, Carolina Scagnolari, Angelo Solimini

**Affiliations:** 1 Dipartimento di Sanitá Pubblica e Malattie Infettive, Sapienza University of Rome, Rome, Italy; 2 Department of Biodiversity and Molecular Ecology, Research and Innovation Centre, Fondazione Edmund Mach, San Michele all'Adige (TN), Italy; 3 Epilab-JRU, FEM-FBK Joint Research Unit, Trento, Italy; 4 Regional Service for Surveillance and Control of Infectious Diseases (SERESMI)—Lazio Region, National Institute for Infectious Diseases "Lazzaro Spallanzani" IRCCS, Rome, Italy; 5 Center for Information Technology, Fondazione Bruno Kessler, Trento, Italy; 6 Department of Molecular Medicine, Laboratory of Virology, Sapienza University of Rome, Rome, Italy; Australian Red Cross Lifelood, AUSTRALIA

## Abstract

Chikungunya virus (CHIKV) is a mosquito-borne infection that is emerging in temperate areas of Europe, following the expansion of one of its vector species, *Aedes albopictus*. Although CHIKV fever is a self-limiting disease, with a clinical syndrome often resolving within few days, it can also cause severe sequelae, including chronic polyarthralgia lasting up to 5 years. Additionally, CHIKV outbreaks may limit blood bank donations, adding economic burden on the health system. Public health authorities in Europe need to increase their preparedness against this emerging threat. Two large CHIKV outbreaks occurred in Italy in 2007 and 2017, with hundreds of cases and significant geographical spread. The aim of this paper is to review and compare the 2 Italian outbreaks in terms of available estimates of key epidemiological features, patient clinical presentation, virus and immunological characteristics, and public health response. Recommendations for public health and future directions for research are also discussed and highlighted.

**Key results:**

Both outbreaks started in small towns, but cases were also detected in nearby larger cities where transmission was limited to small clusters. The time spans between the first and the last symptom onsets were similar between the 2 outbreaks, and the delay from the symptom onset of the index case and the first case notified was considerable. Comparable infection and transmission rates were observed in laboratory. The basic reproductive number (R_0_) was estimated in the range of 1.8–6 (2007) and 1.5–2.6 (2017). Clinical characteristics were similar between outbreaks, and no acute complications were reported, though a higher frequency of ocular symptoms, myalgia, and rash was observed in 2017. Very little is known about the immune mediator profile of CHIKV-infected patients during the 2 outbreaks. Regarding public health responses, after the 2007 outbreak, the Italian Ministry of Health developed national guidelines to implement surveillance and good practices to prevent and control autochthonous transmission. However, only a few regional authorities implemented it, and the perception of outbreak risk and knowledge of clinical symptoms and transmission dynamics by general practitioners remained low.

**Major conclusions:**

Efforts should be devoted to developing suitable procedures for early detection of virus circulation in the population, possibly through the analysis of medical records in near real time. Increasing the awareness of CHIKV of general practitioners and public health officials through tailored education may be effective, especially in small coastal towns where the outbreak risk may be higher. A key element is also the shift of citizen awareness from considering *Aedes* mosquitoes not only as a nuisance problem but also as a public health one. We advocate the need of strengthening the surveillance and of promoting the active participation of the communities to prevent and contain future outbreaks.

## Author summary

In Europe, vector-borne diseases have been increasing during the last decades. CHIKV is an example of a neglected emerging disease transmitted by the alien mosquitoes *Ae*. *albopictus* that caused 2 large outbreaks in Italy in 2007 and 2017. It is important to compare the main epidemiological, clinical, virological, and immunological features, as well as the public health responses, to increase preparedness to face future outbreaks.

## Introduction

Chikungunya virus (CHIKV; genus *Alphavirus*, family Togaviridae) is a positive-stranded RNA mosquito-borne alphavirus that has been causing sustained epidemics in India and Southeast Asia countries in the last decades [1]. Although CHIKV fever is a self-limiting disease, with a clinical syndrome often resolving within few days [2,3], ongoing symptoms (sequelae) can include chronic polyarthralgia that could last up to 5 years affecting patient daily and social life, creating an additional economic burden on the public health system [4,5]. Additionally, CHIKV outbreaks directly affect blood bank donations because blood cannot be drawn from regions of recent virus activity [6]. Following the geographical expansion of one of its vector species, *Aedes albopictus*, several CHIKV outbreaks have been documented in temperate regions [7–12], and many urban areas of Southern Europe have a non-negligible risk of CHIKV outbreaks [13–15]. Therefore, general practitioners and public health authorities need to be prepared to face this emerging arboviral risk [16–18].

Two CHIKV outbreaks occurred in Italy in a 10-year period, being the largest recorded so far in Europe in terms of number of cases and geographical spread. The first outbreak took place in 2007 in Northeast Italy near the Adriatic coast, and it represented the first documented autochthonous CHIKV transmission on continental Europe [9]. In total, 337 cases were notified during 2007, 217 of which were laboratory-confirmed [14,19]. The outbreak started from Castiglione di Cervia and Castiglione di Ravenna and generated smaller transmission chains mainly in 5 other towns in the same region (Emilia-Romagna) [19]. The second CHIKV outbreak occurred in 2017 and was characterized by 3 main foci (Anzio, Rome, and Guardavalle Marina) in 2 different regions, Lazio (Anzio, Rome) and Calabria (Guardavalle Marina), in central and southern Italy [7,8]. In total, the most updated data report 499 probable cases notified during 2017; of these, 270 were then laboratory-confirmed [20].

**Fig 1:**
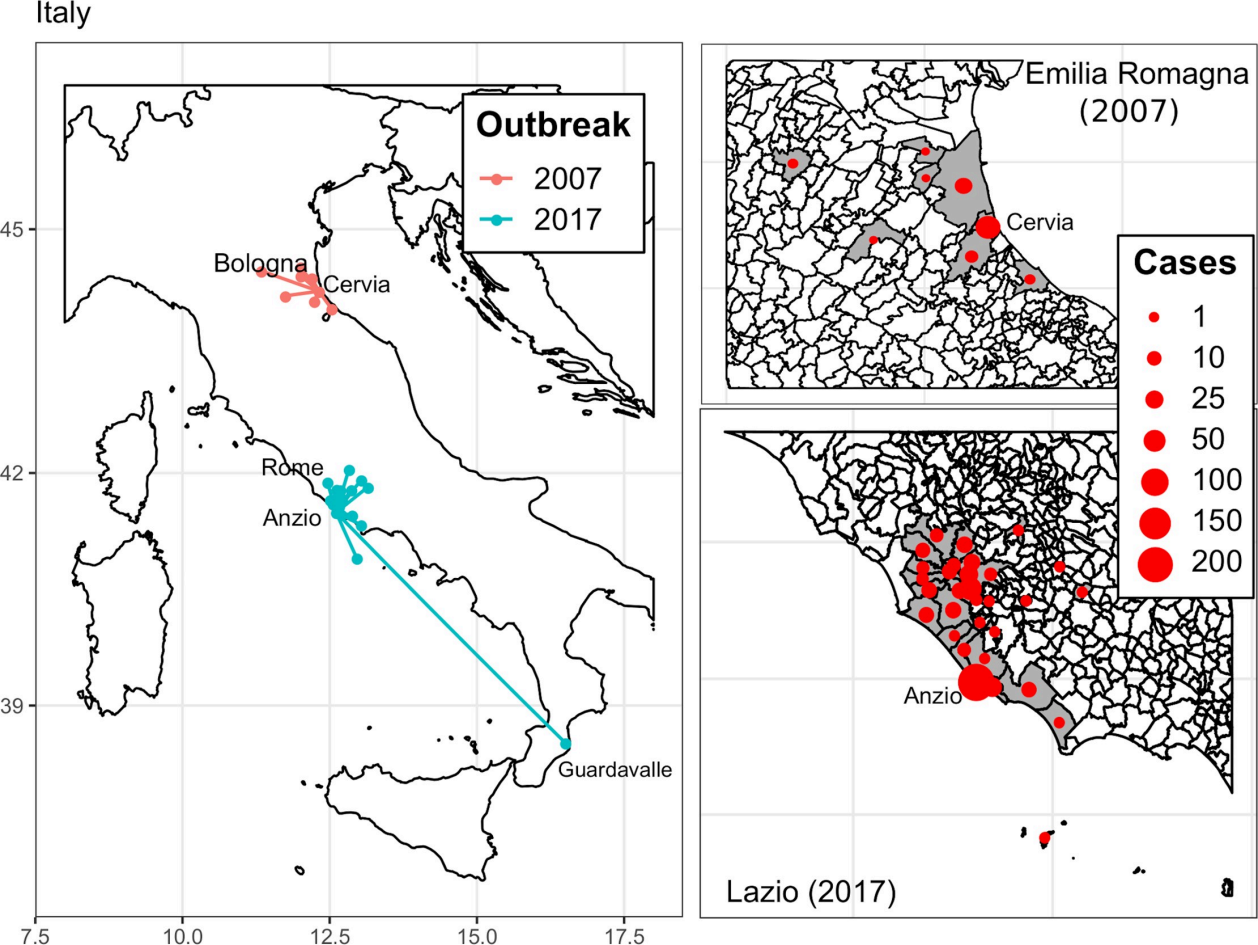
Map of Italian municipalities of residence of human cases during the 2007 and 2017 chikungunya outbreaks. For 2017, 3 cases epidemiologically linked to Anzio but resident in France, Germany, and the Abruzzo region are not displayed. Moreover, 2 more cases were notified in Emilia-Romagna and Marche Region, but no data are available on their municipality of residence [21].

The 2017 outbreak gave rise to epidemiologically linked cases in 16 smaller towns and villages within the Lazio region, in at least 2 other Italian regions (Emilia-Romagna, Marche), and in 2 other European countries (France and Germany) [21] (Fig 1). Interestingly, Rocklöv and colleagues—using Twitter activity data, Google Trends, and Wikipedia page hits to investigate mobility patterns between the 2017 outbreak zones—confirmed the potential for spread between countries and cities in Italy and Europe [22].

Given the importance in terms of number of cases of the 2 Italian outbreaks, useful insights for public health may arise from a comparative analysis of their epidemiological, clinical, and virologic features. The aim of this paper is to review and compare the 2 outbreaks in terms of available estimates of key epidemiological parameters, patient clinical presentation, viral and immunological characteristics, and public health response. Recommendations and future direction for research are also discussed.

## Comparison between 2007 and 2017 Italy outbreaks

### Epidemiological features

Table 1 summarizes the epidemiological parameters measured (or estimated) in the 2 Italian outbreaks. In general, both outbreaks started in small towns, yet small clusters of cases (<10 cases; [19,23]) were detected also in large cities close to the main focal areas (2007: Bologna, 5 cases out of 373,026 inhabitants, 75 km from initial cluster [14], suspected local transmission [24]; 2017: Rome, 80 out of 2,873,486 inhabitants, 62 km from initial cluster [7], confirmed local transmission without epidemiological link with Anzio). The 2017 outbreak had a wider geographical spread compared to the 2007 outbreak (Fig 1), possibly because Anzio is a seaside touristic location with many people commuting to and from Rome or other cities in the region on a daily basis [7]. The index case was identified only in the 2007 outbreak, a resident who traveled from and got infected in Kerala (India) and then visited his cousin (first autochthonous case) in Castiglione di Cervia on 23 June [25].

**Table 1:** Selected epidemiological parameters of the 2007 and 2017 chikungunya outbreaks in Italy.

		2007	2017	
**Infection**	Probable cases	337	499	[19,20]
	Confirmed cases	217	270	[19,20]
	Index case notified	yes	no	[7,9]
**Spread**	N primary foci	2	3	[7,19,48]
	Other municipalities with incident cases	5	16	[7,19,48]
	Spread to other regions	no	yes	[19,21,48]
	Spread to other countries	no	yes	[19,21,48]
**Duration**	Time from first to last autochthonous case	140 days	134 days	[7,19,48]
	First notified symptom onset	4 July	26 June	[7,19,48]
	Last notified symptom onset	20 November	5 November	[7,19,48]
	Index case	23 June	unidentified	[7,19,48]
**Epidemiology**	R_0_ basic reproductive number	range: 1.8–6	range: 1.5–2.6	[6,26–28]
	Attack rate per foci (%)	5.4; 2.5	0.3; 4.3	[7,20,28]
	Vector/host ratio	10–35	1.9–7.3	[6,26,27]
**Vector competence**	Infection rate (%)	range: 40–100	range: 50–100	[30]
	Transmission rate (%)	range: 50–86	range: 75–80	[30]
	Transmission efficiency (%)	41	42	[30]

The time span between the first and the last symptom onset (autochthonous cases) was similar among the 2 outbreaks, being 140 days in 2007 (from 4 July 2007 to 20 November 2007) and 134 days in 2017 (from 26 June 2017 to 5 November 2017) [7,9]. During both outbreaks, the delay observed from the beginning of virus local transmission to the first notification was considerable: 37 days (9 August 2007, first warning from a citizen; 14 August 2007, first list of suspected cases identified through active case search [25]) and 75 days (7 September 2017, first 3 potential cases notified by serum and urine samples; [8]).

Despite the public health relevance of such outbreaks, only a few mathematical models have been applied to characterize CHIKV transmission dynamics [6,26]. For the 2007 outbreak, the available estimates of the basic reproductive number (R_0_) were in the range of 2–2.3 with mean value 2.15 [27], 3.4–4.6 with mean value 4 [28], and 1.8–6 with mean value 3.3 [26]. The differences in R_0_ estimates are primarily due to different modeling methodologies and parameters (e.g., vector susceptibility and/or longevity). Only one study estimated R_0_ for the 2017 outbreak, resulting in the range 1.5–2.6 with mean value 2 [6] (see Table 1).

The cumulative incidence of notified cases per 100,000 residents were 335.1 in Anzio, 4,263 in Guardavalle Marina, from 1.6 to 13.9 in Rome [7], 5,389 in Castiglione di Cervia, 2,508 in Castiglione di Ravenna, and 1.3 in Bologna [9]. A seroprevalence study found that 10.2% of the population in Castiglione di Cervia and Castiglione di Ravenna had been exposed to CHIKV in 2007 [29]; similar proportions were estimated in Poletti and colleagues [26]. No results from serosurveys are yet available for the 2017 outbreak.

Due to the lack of entomological surveillance, vector abundance was estimated retrospectively using entomological records. Regarding the 2007 outbreak, 9.2 biting females per human per day were estimated (with a population density of 25.2 persons per hectare in the study area) using 2008 human landing capture (HLC) experiments [27], while 10 to 35 female mosquitoes per human during the peak of mosquito abundance were estimated using data from ovitraps in 2008 [26]. A lower mosquito/human ratio was estimated in Lazio at the time of the first symptom onset in 2017, with the ratio ranging between 1.9 and 7.3 in coastal sites and between 0.4 and 2.6 in urban areas [6]. The latter estimates were obtained by using a mathematical model similar to the one adopted in [13] but calibrated on captured female adult mosquitoes during 2012. To the best of our knowledge, no other measures of vector abundance have been reported so far.

Estimates of vector competence have been computed for both viruses involved in the 2007 and 2017 outbreaks. The results, available in Fortuna and colleagues [30], showed a comparable infection and transmission rate for both lineages. Infection and transmission rates were defined as number of positive bodies/total fed females and as number of positive saliva/positive bodies, respectively. Batches of 10 mosquitoes were processed for each viral strain at 0, 3, 14, and 20 days postexposure. In 2007, infection rates ranged from 100% (day 0) to 40% (day 20) while transmission rates ranged from 86% (day 3) to 50% (day 14). In 2017, infection rates ranged from 100% (day 0) to about 50% (day 3), while transmission rates ranged from 75% (day 3) to 80% (day 14). Interestingly, the transmission efficiency computed as number of positive saliva/total fed females was almost identical for both viruses, 41% (2007) and 42% (2017) [30].

### Clinical presentation of patients and public health impact

During the 2 CHIKV outbreaks, no cases among pregnant women were reported, and 1 death per each outbreak was registered, both being old patients (77 and 83 years old, respectively) affected by severe underlying medical conditions. Females were slightly more affected than males in both outbreaks (52% in 2007, 54.2% in 2017), and, although the use of different age groups may limit the comparison, no main differences for patient’s age distribution were observed [7,9]. Concerning the patient’s clinical characteristics, data collected during the 2 Italian CHIKV outbreaks were related to confirmed cases in 2007 [9] and to probable and confirmed cases in 2017 [7] (Table 2). Overall, clinical characteristics were similar between outbreaks, though a higher frequency of ocular symptoms, myalgia, and rash was observed in 2017 (Table 2). Finally, concerning hospital admission related to CHIKV infection, data were available only for the 2017 outbreak, when 9.4% (*n* = 35) of CHIKV cases were hospitalized because of their clinical condition [7]. No acute complications were reported during 2007 and 2017 CHIKV outbreaks [7,9].

**Table 2:** Clinical characteristics of CHIKV infection outbreak in Italy (2007 and 2017). Data from [7,9].

Clinical Symptom or Sign	2007 (Emilia-Romagna; *n* = 205)*		2017 (Lazio, *n* = 402)**	
	*n*	%	*n*	%
Fever	205	100%	389	96.8%
Arthritis	–	–	159	39.6%
Arthralgia	199	97%	385	95.8%
Headache	105	51%	206	51.2%
Myalgia	94	46%	254	63.2%
Retro-orbital pain/photophobia	31	15%	49	12.2%
Conjunctivitis	7	3%	60	14.9%
Rash	106	52%	253	62.9%
Asthenia	190	93%	311	77.4%
Diarrhea	48	23%	–	–
Vomiting	40	19%	–	–

*Confirmed cases only.

**Confirmed (*n* = 200) and probable (*n* = 202) cases.

**Abbreviations:** CHIKV, chikungunya virus.

During 2017, Italy experienced also a large spread of measles, with almost 5,000 cases reported, of which 1 out of 3 cases were in the Lazio region (incidence rate: 28.8 cases/100,000 inhabitants). It should be noticed that CHIKV-related skin rash is usually morbilliform (measles-like) [31–33], with or without acral and facial edema, mucosal, and genital and intertriginous ulceration, and vesiculobullous eruptions are more likely to occur in children. Differently from measles, after an initial facial flushing, the face is often spared by the CHIKV-related exanthema [31]. Therefore, it is possible to speculate that some CHIKV cases with nonsevere joint involvement in the acute phase of infection may have been clinically underrecognized, and thus under-reported.

A common postacute complication is a chronic and disabling joint pain, usually lasting for few months to up to 3 years [3]. A prospective longitudinal study conducted after the 2007 outbreak [34] showed that, after 1–3 years from acute CHIKV infection, one-third of patients complain of arthralgia, frequently associated with reduced functional ability, with episodic relapse and recovery periods. Chronic arthralgia is a frequent complication of acute CHIKV disease with a significant long-lasting reduction of functional ability, adding costs to the public health system beyond the strict outbreak time period. A recent cost-effectiveness analysis has shown that in Italy, the average cost of illness per CHIKV case was €424.9 (95% CI: 280.4, 795.5), and its relative burden in terms of Disability Adjusted Life Years (DALYs) was 0.45 years (95% CI: 0.01, 2.57) [5]. Additionally, the public health impact of arboviral infection, including CHIKV infection, is particularly relevant for the safety of the blood-transfusion system, especially during the outbreak. Precautionary measures applied in Italy to regions where CHIKV infection occurred include a 3–4 weeks deferral of donors who stayed (even for a short-time) in the geographical areas affected, as well as a 4-week deferral after the resolution of symptoms for donors who were diagnosed with CHIKV infection [35]. In comparison to the 2007 outbreak, the 2017 outbreak involved a large city (the municipality of Rome), and its impact on the blood-transfusion system was greater [6,35]. The application of quarantine based on active recall of all donors and additional inactivation procedure for platelet concentrates caused significant economic and logistic impacts on the management of the blood-transfusion system at both the local and national level [36].

### Viral characteristics and immunological response

The 2007 outbreak was caused by a newly emerged Indian Ocean Lineage (IOL) variant characterized by an amino acid (aa) shift (A→V) at the 226 position of the membrane fusion glycoprotein E1. This E1–A226V mutation was appeared independently multiple times, including the 2005–2006 Indian Ocean CHIKV outbreak and improved replication and transmission efficiency of CHIKV in the *Ae*. *albopictus* population from Réunion Island [37]. However, recent experimental CHIKV infections of the Italian *Ae*. *albopictus* population showed a similar vector competence for both viral strains, with and without A226V mutation [30].

Also, the 2017 outbreak was caused by the East Central South African (ECSA) genotype IOL strains, but both human and mosquito strains isolated in 2017 did not carry the E1–A226V mutation [7,38]. Moreover, E1 gene sequences from the patients and from the mosquitoes were identical and very similar to the CHIKV strain involved in a recent epidemic in Pakistan [8,39] and most likely introduced into Italy in May 2017.

Little is known about the immune mediator profile of CHIKV-infected patients during the Italian 2007 and 2017 outbreaks and whether the immunological signature has changed according to the IOL variant with or without the E1–A226V mutation. The potential contribution of cytokines to disease has been reported in both the 2007 and 2017 outbreaks. Specifically, in the 2007 outbreak, the severity of CHIKV disease was associated with increased levels of Chemokines ligand 9 ((CXCL9/ Monokine induced by gamma interferon MIG), CXCL10/IP-10, and immunoglobulin G (IgG) [40]. At the same time, a remarkable abnormal pattern of circulating cytokines—interferon (IFN)-α, IFN-β, and interleukin (IL)-6—was found in a unique lethal CHIKV case during the 2017 outbreak, which involved an elderly patient with underlying cardiac disease [41]. All these findings underline the key role played by cytokines in controlling viral replication and pathogenesis during the early stages of CHIKV infection, suggesting that well-balanced immune responses are crucial for early containment of CHIKV infections. However, beside the multiple host factors involved in the activation of immune response to viral infection, CHIKV has developed mechanisms to evade early cellular immunity by, e.g., circumventing the antiviral activity of type I IFNs [42]. Interestingly, the CHIKV IOL strain with the A226V mutation obtained from patients during the epidemic of 2007 in Italy were more sensitive to type I IFNs compared to the viral strains without A226V, suggesting that level of resistance of the CHIKV to the antiviral action of IFN could actually be virus-strain dependent [43,44].

### Public health response

After the 2007 outbreak in Castiglione di Ravenna, the Italian Ministry of Health established a multidisciplinary team for developing national guidelines to implement *Aedes* surveillance as well as guide good practices to prevent and control autochthonous transmission. Following the European Centre for Disease Prevention and Control (ECDC) guidelines [45], the Italian Public Health authorities developed 3 different epidemiological risk scenarios according to the presence of Invasive Mosquito Species (IMS) and infected cases. The 3 epidemiological scenarios were i) locally established IMS with no arbovirus infection, ii) high abundance of IMS and presence of imported arbovirus cases, and iii) high abundance of IMS with 1 autochthonous or cluster cases. Different surveillance and control procedures according to the identified risk were defined [21]: i) vector surveillance using ovitraps in the locality where the presence of mosquitoes, as well as entry points (e.g., ports, airports) and possible routes of spread, were confirmed and pest control in the municipality using larvicides in manholes; ii) vector surveillance to assess the relative abundance and seasonality of adult mosquitoes, as well as insecticide spraying and the removal of breeding sites by carrying out door-to-door activities around 200 m of imported cases; iii) vector surveillance, screening of pathogens, and removal and treatment of larval breeding sites to target mosquito elimination through an area of 200 m around the infected cases; and iv) assessment of efficacy of control measures.

Mostly because of the lack of resources, only few regional public health authorities had the opportunity to implement the guidelines regarding surveillance activities. The region where the 2007 outbreak took place (Emilia-Romagna) implemented an *Aedes* monitoring system by means of ovitraps and a massive citizen information campaign (http://www.zanzaratigreonline.it/). Few other regions implemented similar measures (mostly in northeastern areas of the country), and the perception of risk of outbreaks by general practitioners (GPs) remained still low. A questionnaire based survey on knowledge, attitude, and practices (KAP), carried out in Rome in summer 2012, found that less than one-third of GP responders correctly identified the CHIKV-endemic countries, ways of transmission, major symptoms, duration of the incubation period, and long-term complications and were aware of specific preventive initiatives led by health authorities [16]. Those results suggest that information campaigns and activities that were carried out after the 2007 outbreak were not sufficient to build up the GPs or citizens awareness toward CHIKV—at least, in the city of Rome.

The lack of awareness and knowledge of CHIKV may partially explain the late detection of the 2017 outbreak. In fact, late May was estimated as the most probable period of introduction, while the first notified case was dated September 7 [6]. Additionally, the 2017 outbreak highlighted the risk of CHIKV transmission in many coastal areas [6], although large cities like Rome might also be at risk of autochthonous transmission [15].

## Outlook and future research needs

Given the importance of CHIKV as emerging disease threat, efforts should be devoted to developing suitable procedures for early detection of virus circulation in the population, possibly through analysis of medical records in near real time. The most frequent symptoms of CHIKV fever and joint pain are often associated with other common diseases and go unnoticed. Increasing the awareness of CHIKV of GPs and public health officials through tailored education may be effective, especially in small coastal towns where the outbreak risk is higher. A key element is also the shift of citizen awareness from considering *Aedes* mosquitoes not only as a nuisance problem but also as a public health one. Nowadays, because of the lack of CHIKV vaccines, a key role in the prevention of CHIKV outbreaks is played by the participation of the community in environmental management [46]. Specifically, citizens should be educated in how to reduce *Ae*. *albopictus* breeding sites, also with the support of trained scientific staff inspecting potential vector habitats in private premises [47]. Moreover, novel data sources should be considered for inclusion in the surveillance systems of both IMS and pathogens. Finally, the reasons why diseases such as CHIKV and dengue are causing sporadic but repeated epidemic events in temperate climate areas should be further investigated.

**Key learning points**

- The main epidemiological parameters (e.g., infection and transmission rates, basic reproductive numbers, and others) and patient clinical presentation were very similar between the 2 outbreaks, although in 2017, a larger geographical spread of cases was recorded.
- During the 2017 outbreak, cases were reported in the metropolitan area of Rome, and its impact on the blood-transfusion system was greater than the 2007 outbreak, when cases were reported only in smaller towns.
- To prevent and contain future outbreaks, there is an urgent need to strengthen the surveillance system and to promote the active participation of the communities.

**Top 5 papers**

1. Rezza G, Nicoletti L, Angelini R, Romi R, Finarelli A, Panning M, et al. Infection with chikungunya virus in Italy: an outbreak in a temperate region. Lancet. 2007;370: 1840–1846.
2. Vairo F, Mammone A, Lanini S, Nicastri E, Castilletti C, Carletti F, et al. Local transmission of chikungunya in Rome and the Lazio region, Italy. Roques P, editor. PLoS ONE. 2018;13: e0208896.
3. Burt FJ, Chen W, Miner JJ, Lenschow DJ, Merits A, Schnettler E, et al. Chikungunya virus: an update on the biology and pathogenesis of this emerging pathogen. Lancet Infect Dis. 2017;17: e107–e117.
4. Carletti F, Marsella P, Colavita F, Meschi S, Lalle E, Bordi L, et al. Full-Length Genome Sequence of a Chikungunya Virus Isolate from the 2017 Autochthonous Outbreak, Lazio Region, Italy. Genome Announc. 2017;5: e01306-17.
5. Ng LFP. Immunopathology of Chikungunya Virus Infection: Lessons Learned from Patients and Animal Models. Annu Rev Virol. 2017;4: 413–427.
